# Smile esthetic evaluation of mucogingival reconstructive surgery

**DOI:** 10.1007/s10266-020-00544-6

**Published:** 2020-08-09

**Authors:** Roberto Rotundo, Luigi Genzano, Michele Nieri, Ugo Covani, David Peñarrocha-Oltra, Miguel Peñarrocha-Diago

**Affiliations:** 1 Honorary Associate Professor in Periodontology, UCL Eastman Dental Institute, London, UK; 2 Private Practice, Prato, Italy; 3 Department of Experimental and Clinical Medicine, University of Florence, Florence, Italy; 4 Istituto Stomatologico Toscano, Camaiore, Italy; 5 Valencia University Medical and Dental School, Valencia, Spain; 6 Via Vasco de’ Gama 33/3, 50127 Florence, Italy

**Keywords:** Smiling, Esthetics, Gingiva, Gingival recession, Mucograft, Clinical trials

## Abstract

To assess the difference in smile esthetic impact of Coronally Advanced Flap (CAF) with or without the adjunct of a collagen matrix (CMX) used as root coverage procedures. Subjects with esthetic demands showing multiple upper gingival recessions of at least 2 mm, without interproximal attachment loss and cervical abrasion no more than 1 mm were recruited and randomized to CAF plus CMX or CAF alone. The Smile Esthetic Index (SEI) was adopted to quantify the quality of the smile recorded at baseline and 12 months after treatment for each treatment group. In addition, between group difference in the SEI was calculated. 24 Patients were treated and analysed. At baseline, mean gingival recession depths were 2.3 ± 0.7 mm for Test group and 2.6 ± 1.0 mm for Control group. After 1 year, the residual recession depth was 0.3 ± 0.4 mm in the CAF + CMX group and 0.6 ± 0.3 mm in the control group. The SEI at baseline was 8.1 ± 1.0 and 7.9 ± 0.7 for Test and Control group, respectively. The between groups difference at 12 months in SEI was 0.4 (95% C.I. **− **0.0 to 0.8, *P* = 0.0697). Twelve months after treatment, CAF + CMX provided a similar SEI compared to CAF alone and the adjunct of a collagen matrix did not show a different impact on the smile esthetic appearance.

## Introduction

A single-mode survey of dental practices carried out by the American Academy of Cosmetic Dentistry (AACD) in 2015 indicated that 86% of patients elect cosmetic treatments to improve physical attractiveness and self-esteem [1]. In particular, appearance was indicated by 99% of the participants as the top priority, followed by cost (85%) and longevity of treatment results (79%).

Several studies have reported the smile esthetics as a dominant concern for patients and able to positively influence attractiveness [2, 3]. The mouth and thus the teeth were also investigated in Psychology as important factors in the evaluation of attractiveness [4, 5]. Other authors reported that teeth are the second most important facial feature when assessing beauty, after the eye [6]. More recently, the Smile Esthetic Index (SEI) has been suggested as a reliable and validated method to measure the esthetic impact of a smile [7, 8].

However, little information is provided by the literature about the relationship between esthetics of the smile and gingival recessions. Some indications come from a Swiss study, where the authors investigated the indications for the treatment of gingival recessions through a questionnaire administered to 3780 dentists, representing over 95% of all dentists working in Switzerland [9]. Results showed that esthetic concerns were the predominant indication for root coverage procedures, and therefore, future researches should include esthetic aspects as primary clinical outcome variables. In addition, Rotundo et al. [10] reported data indicating that only complete root coverage is actually perceived as the most successful outcome by patients, dentists, and periodontists.

Among the proposed surgical procedures, the Coronally Advanced Flap (CAF) showed one of the highest performance level for treating single and multiple recessions in terms of esthetic results and patients’ morbidity. Nevertheless, the combination with autologous connective tissue graft achieves even higher percentages of complete root coverage (CRC) in cases of gingival recessions without interproximal attachment loss and non-carious cervical lesions, with long term stability [11, 12]. Meanwhile, the use of soft tissue substitutes (STS) in mucogingival surgery revealed interesting results due to its easier and less invasive approach. At the same time, several investigations focused their interest to test not only the safety but also the efficacy of the proposed newly formed materials when compared to soft tissue autografts [13–16]. In particular, the use of collagen matrix xenograft (CMX) for root coverage procedures has shown positive clinical effects and, actually, it can be considered as a valid alternative to the CTG [17–19]. In particular, Tonetti et al. recently compared the adjunct of a xenogeneic collagen matrix or connective tissue graft to coronally advanced flaps for treating multiple adjacent gingival recessions by means of a multicenter study. Data shown that in terms of complete root coverage, at 6 months, the probability to obtain complete root coverage was significantly higher for CTG group than CMX cases [16].

In another single-centre, superiority, assessor-blind clinical trial [20], CAF was tested in combination with CMX and compared to CAF alone. Results reported at 1 year showed similar clinical performances in terms of root coverage compared to CAF alone, but with the only significant difference in terms of gingival thickness in favour of CMX group. Patient-related outcomes and measures from this study revealed similar esthetic results, recorded as patient’s judgment of the treated sites (VAS), with no statistical differences between the 2 treated groups.

However, no data have been discussed in the current literature about the influence that such a material might have on the esthetic outcome of the treated tissues and if, a scar-like appearance of the area may result at the end of treatment.

Therefore, the aim of this study was to evaluate the difference in terms of esthetic of the smile using the Smile Esthetic Index between CAF plus CMX and CAF alone performed to treat multiple adjacent gingival recessions.

## Materials and methods

### Study design

The present study reports secondary outcomes of a previous single-centre randomized controlled trial with two parallel groups design [20]. The study protocol was approved by the local ethical committee (prot. 24/CESM). All study participants signed a proper informed consent in agreement with the Declaration of Helsinki on experimentation involving human subjects.

### Participants

Subjects afferring to a private office in Italy were considered for the study and defined eligible if the following criteria were satisfied: (1) 18 years or older; (2) presence of at least 2 upper adjacent teeth affected by at least 2 mm depth gingival recessions with identifiable cemento-enamel junction (CEJ) (non-carious cervical lesions < 1 mm of depth); (3) good oral hygiene level; (4) Full Mouth Plaque (FMPS) and Bleeding (FMBS) Score < 20%. The following main exclusion criteria were considered before the commencement of the study: (1) smoking habit; (2) pregnant status; (3) uncontrolled diabetes; (4) absolute contraindications for surgical treatment; (5) radiotherapy or chemotherapy for malignancy within the past 5 years; (6) medications or treatments able to impair mucosal wound healing; (7) systemic conditions altering connective tissue metabolism; (8) body reactions to collagen materials; (9) presence of active periodontal disease; and (10) participation in another clinical trial in the last 6 months.

### Treatment phase

After the screening phase, a single calibrated examiner (LG) provided a periodontal examination to all patients considered eligible for the study. In particular, type of tooth, Full Mouth Plaque (FMPS) and Bleeding Score (FMBS), gingival recession depth (Rec), keratinized tissue width (KTw), gingival thickness (GThick), and pocket depth (PD) were recorded.

In addition, a questionnaire was administered to assess the esthetic condition and the overall satisfaction of each patient, at baseline and 1 year after treatment.

The enrolled patients were then instructed on proper home dental hygiene procedures, paying attention to correct traumatic toothbrushing habits, and an initial phase of professional supragingival scaling and polishing was performed.

A single calibrated operator (RR) performed all surgical interventions. After initial local anesthesia, an envelope flap without releasing incisions was performed [21]. The incisions were mesially and distally extended to include one tooth more on each side of the interested area. In correspondence of the interdental area, oblique and bevelled surgical incisions were performed to elevate an initial split thickness flap in the papillae area. The flap was then raised as full-thickness till to the mucogingival line to preserve all the residual keratinized tissue. All the interdental papillae were now de-epithelialized and the exposed root surfaces, with the exception of a 1 mm connective attachment area close to the bone crest, were mechanically debrided. Afterward, the last portion of the flap was elevated in a split-thickness mode by means of a single and linear incision into the vestibular lining mucosa to detach the flap from the deeper muscle insertions. In this moment, the randomized allocation of the patient was revealed by means of opening the sealed envelope. A collagen matrix (Geistlich Mucograft^®^, Geistlich Pharma AG) was used as connective tissue substitute and placed onto all exposed roots following the manufacturer’s instructions. In particular, the matrix was trimmed, and blocked in correspondence of the exposed roots by means of single interrupted resorbable sutures anchored at the base of each interdental papillae. The substitute was then spontaneously embedded by the blood. After that, the flap was coronally sutured to the cemento-enamel junction with sling resorbable sutures, paying particular attention to avoid any compression of the matrix. In the control group, the flap was sutured immediately after the split thickness flap was perfumed by means of sling sutures, as previously described for the test group.

At the end of surgical phase, patients were instructed to avoid any traumatic movement of the area, including the toothbrushing procedures, for the initial 3-week period. A chlorhexidine mouth rinse (0.12%) was prescribed for 3 weeks (twice a day), and an anti-inflammatory therapy (Ibuprofen 600 mg) suggested according to individual needs. Sutures were removed after 14 days.

### Outcome assessment

The main objective of this study was to objectively measure and compare the esthetic of the smile of patients treated with CAF + CMX (test group) or CAF alone (control group) affected by upper multiple adjacent gingival recession. This data was then compared with subjective judgment of the same patients recorded in a previous analysis [20].

### Sample size

Considering a 1 mm difference in gingival recession reduction between study groups (standard deviation of 0.93 mm) [21] with a two-side 5% significance level, a power of the study set at 90%, the requested minimum number of participants per group was 12, with a total of 24 subjects needed for the study execution.

Details of the study protocol and clinical results including the primary and secondary outcomes were presented in a previous paper [20].

### Esthetic assessment

#### Objective esthetic assessment

The *Smile Esthetic Index (SEI)* is a validated method to objectively measure the esthetics of a smile [7]. Ten clinical variables were used for this purpose: *smile line*, *facial midline, tooth alignment, tooth deformity, tooth dyschromia, gingival dyschromia, gingival recession, gingival excess, gingival scars, diastema/missing papilla*. The index is valid only on smiles showing all teeth, and the absence of teeth represents criteria of not application for the index.

**Fig. 1:**
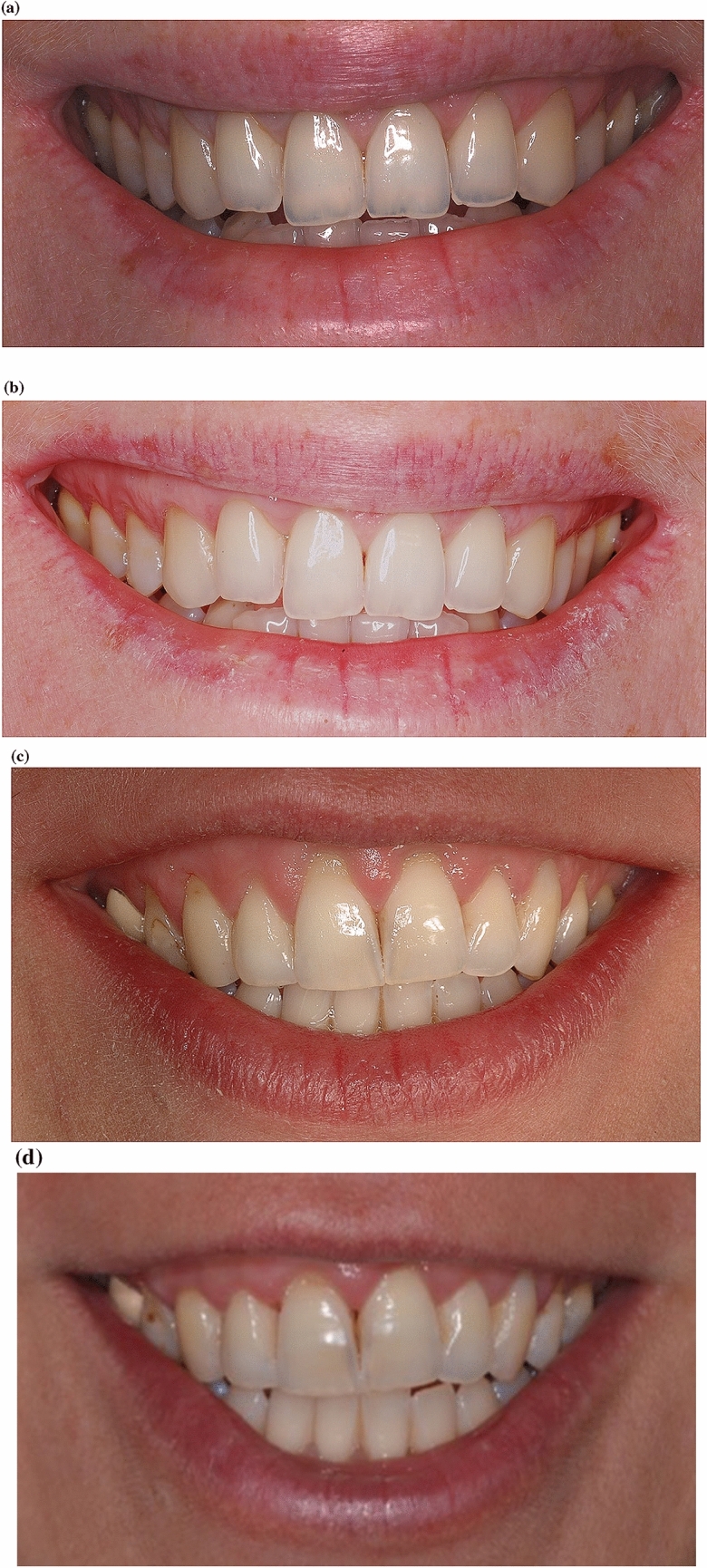
**a** Picture of a frontal smile at baseline—coronally advanced flap group. **b** Picture of a frontal smile at end of follow-up—Coronally Advanced Flap group. **c** Picture of a frontal smile at baseline—Coronally Advanced Flap + Collagen Matrix Xenograft. **d** Picture of a frontal smile at end of follow-up—Coronally Advanced Flap + Collagen Matrix Xenograft

**Fig. 2:**
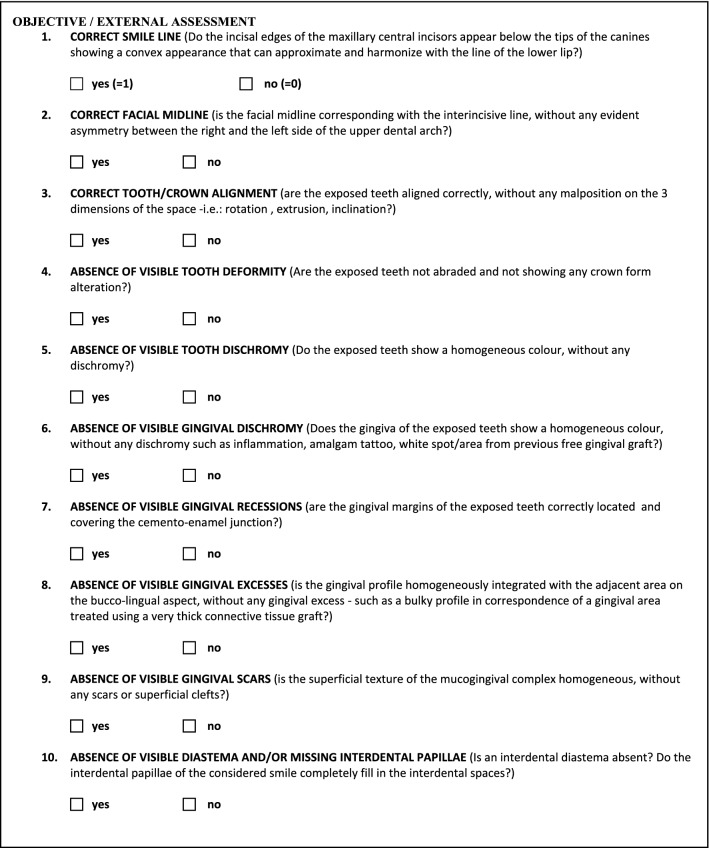
Worksheet adopted for the objective assessment of the smile. The Smile Esthetic Index (SEI)

An assessment worksheet was created and filled in after a detailed and deep analysis of the frontal pictures of a natural smile of each patient taken during a spontaneous speech (Fig. 1a–d). A single independent examiner (DP) performed all assessments. The scores 1 or 0 were attributed considering the presence or absence of the considered variable, respectively. In detail, the value *1* was given in case the variable was present in the analysed picture; the same value was attributed when the variable was not visible within the exposed smile (i.e., gingival recessions not visible in a smiling frontal picture), meaning that it was not able to influence the quality of the exposed smile. The value *0* was given when the considered variable was not correctly represented. At the end, adding all the obtained scores from each variable assessment, the final number represented the Smile Esthetic Index (SEI) of that patient. The worksheet used for the analyses of the considered smiles is reported in Fig. 2.

#### Subjective esthetic assessment

At 12 months after surgery, a linear visual analogue scale was used to assess the patient esthetic concerns related to their smile, asking to each participant to give a judgment, from 0 (corresponding to a *perfect smile*) to 10 (corresponding to a *very bad smile*).

## Data analysis

A descriptive statistics, with mean and standard deviation for quantitative data and frequency and percentage for qualitative data, was performed. For the SEI difference between baseline and 12-month follow-up, an analysis of covariance (ANCOVA) was applied using *treatment* as an explicative variable and *SEI at baseline* as a covariate.

For the 12-month follow-up esthetic VAS, the *t test* was used. Estimates for the treatment effect, standard errors, *p* values and 95% confidence intervals were also provided. The JMP 13.0.0 (SAS Institute Inc.) was used as statistical software.

## Results

**Fig. 3:**
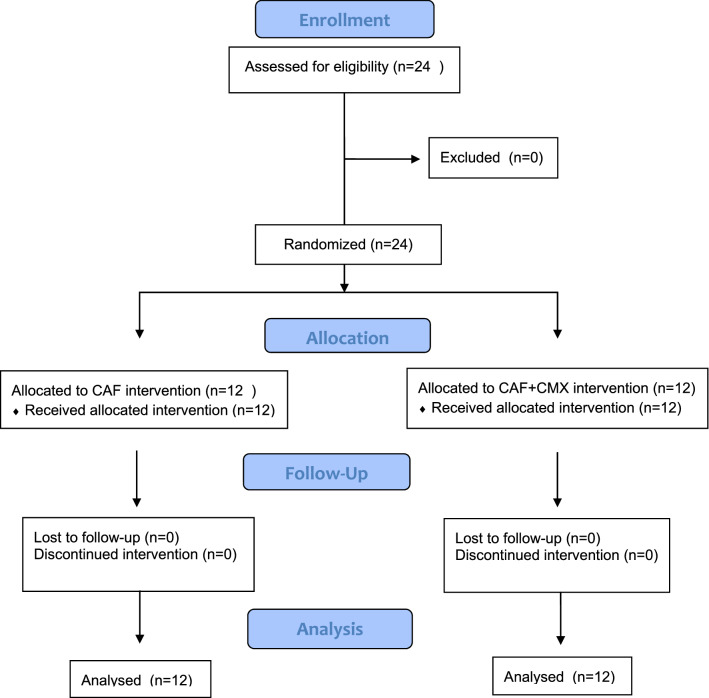
Consort flow diagram

Twenty-four patients (61 gingival recessions) were recruited and treated according to the inclusion criteria (Fig. 3). Descriptive statistics at baseline is reported in Table 1.

**Table 1:** Baseline descriptive statistics

Variable	CAF N = 12 Pat	CMX N = 12 Pat
Age (years)	38.1 (7.3)	31.4 (4.9)
Gender (% Female)	10 (83%)	9 (75%)
Recession (total)	31	30
Incisor	3 (10%)	3 (10%)
Canines	10 (32%)	8 (27%)
Premolars	17 (55%)	17 (57%)
Molars	1 (3%)	2 (7%)
Recession depth (CEJ-GM) mm	2.6 (1.0)	2.3 (0.7)
Gingival thickness (mm)	1.5 (0.6)	1.4 (0.7)
Keratinized tissue width (mm)	3.5 (1.8)	3.3 (1.5)
Full mouth plaque score (%)	0 (0%)	0 (0%)
Full mouth bleeding score (%)	0 (0%)	2 (7%)
Pocket depth mm	1.5 (0.5)	1.5 (0.5)
Clinical attachment loss mm	4.2 (1.3)	3.8 (0.6)
Smile esthetic index	7.9 (0.7)	8.1 (1.0)

Considering the *objective* esthetic outcomes recorded by means of SEI, at baseline the values were 8.1 ± 1.0 and 7.9 ± 0.7 for Test and Control group, respectively. The difference between baseline and end of follow-up (1 year) was 0.7 ± 0.5 for Test group and 0.3 ± 0.5 for the Control group, with a difference between groups of 0.4 (95% C.I. − 0.0 to 0.8, *P* = 0.0697).

Considering the *subjective* esthetic outcomes recorded by means of VAS, outcomes from previous study showed a mean value of 9.3 ± 1.0 for test group, and a mean value of 8.8 ± 2.0 for the control group. Only one patient belonging to the CAF group was not available at 1-year follow-up for recording its esthetic satisfaction. The calculated difference not statistically significant was 0.4 (95% C.I. − 0.9–1.8, *P* = 0.5094). Results from inferential analysis are reported in Table 2.

**Table 2:** Inferential statistics (*t* tests) on patient-reported outcomes and experienced measures

Variable	CAF *N* = 12	CMX + CAF *N* = 12	Difference	95%CI	*P* value
Satisfaction VAS (1 year)	9.1 (1.6)*	9.3 (1.5)	0.2	− 1.1; 1.6	0.7092
Esthetics VAS (1 year)	8.8 (2.0)*	9.3 (1.0)	0.4	− 0.9; 1.8	0.5094
SEI (1 year)	0.3 (0.5)	0.7 (0.5)	0.4	− 0.0 to 0.8	0.0697

*N = 11

## Discussion

Teeth, gingival scaffold, and lip framework are the primary components of a smile. The interaction between these factors requires an individual analysis [23].

Specifically, facial, periodontal, and tooth-related factors such as facial midline, smile line, tooth shape, tooth deformity, tooth alignment and absence of diastema seems to be the factors mainly perceived by the patients [24–26]. In particular, the gingival display represents an important factor influencing the smile. Furthermore, the shape and position of the free gingival margins, the colour of the gingiva, the presence/absence of interdental papillae, the gingival excesses and/or the gingival recessions are determinant factors for soft tissue esthetics [27].

For the time being, the only validated method available to objectively quantify the esthetic value of a smile is the Smile Esthetic Index, consisting on the assessment of 10 different variables recorded in a specific worksheet. The presence/absence of the aforementioned variables corresponds to a number (0 or 1), and the sum of the attributed numbers represents the SEI of that subject (from 0, very bad - to 10, very good) [7].

The aim of the present analysis was to assess the objective esthetic impact on smiles of patients treated with 2 different root coverage procedures (CAF with or without the adjunct of a CMX) using SEI and no differences were identified between the 2 groups (*P* = 0.0697), indicating that the adjunctive use of a CMX to CAF is not able to negatively affect the esthetics of the smile.

Also from the subjective point of view, no differences were observed when a VAS was used to calculate the esthetic impact of these 2 different surgical approaches for treating multiple gingival recessions. These results are in line with the ones reported on single recessions by Stefanini et al. [28].

Based on our results, both groups showed a similar trend and in line each other in objective and subjective smile assessments. In other words, the esthetic evaluation of the treated area given independently by the patients followed the same positive improvement showed by the objective assessment obtained by means of SEI.

Limitations of the present study might be represented by the reduced number of treated patients, with a relatively shallow gingival defects (mean recession depth of 2.5 mm). However, the quality of methodology, the appropriate sample size calculation and related statistical analysis, the active phase performed by a single experienced operator, followed by a different well calibrated examiner, and the absence of drop-outs during the follow-up represent the main strengths of the trial.

In conclusions, based on the results on the present investigation, the subjective and objective smile esthetic assessment of patients treated with CAF associated with a xenogenic collagen matrix provided similar results to CAF alone 1 year after surgery.
